# Cytokine Tuning of Intestinal Epithelial Function

**DOI:** 10.3389/fimmu.2018.01270

**Published:** 2018-06-05

**Authors:** Caroline Andrews, Mairi H. McLean, Scott K. Durum

**Affiliations:** ^1^Cancer and Inflammation Program, Center for Cancer Research, National Cancer Institute, National Institutes of Health, Frederick, MD, United States; ^2^School of Medicine, Medical Sciences and Nutrition, University of Aberdeen, Aberdeen, United Kingdom

**Keywords:** chemokine, cytokine, epithelium, intestine, mucosal immunology

## Abstract

The intestine serves as both our largest single barrier to the external environment and the host of more immune cells than any other location in our bodies. Separating these potential combatants is a single layer of dynamic epithelium composed of heterogeneous epithelial subtypes, each uniquely adapted to carry out a subset of the intestine’s diverse functions. In addition to its obvious role in digestion, the intestinal epithelium is responsible for a wide array of critical tasks, including maintaining barrier integrity, preventing invasion by microbial commensals and pathogens, and modulating the intestinal immune system. Communication between these epithelial cells and resident immune cells is crucial for maintaining homeostasis and coordinating appropriate responses to disease and can occur through cell-to-cell contact or by the release or recognition of soluble mediators. The objective of this review is to highlight recent literature illuminating how cytokines and chemokines, both those made by and acting on the intestinal epithelium, orchestrate many of the diverse functions of the intestinal epithelium and its interactions with immune cells in health and disease. Areas of focus include cytokine control of intestinal epithelial proliferation, cell death, and barrier permeability. In addition, the modulation of epithelial-derived cytokines and chemokines by factors such as interactions with stromal and immune cells, pathogen and commensal exposure, and diet will be discussed.

## Introduction

The intestinal epithelium separates the diverse and ubiquitous members of the intestinal luminal microbiome, virome, and mycobiome from the largest population of resident immune cells anywhere in the body, forming our largest single barrier to the external environment (1–4). As such, in addition to its critical role in digestion, the gut epithelium is also charged with mediating much of the interaction between luminal organisms and immune cells to ensure appropriate defensive reactions to pathogens versus tolerance of commensal microorganisms (1).

The physical intestinal barrier consists of a continuous single layer of columnar epithelial cells overlain by a variably thick layer of mucus. This mucus layer is embedded with antibodies and antimicrobial peptides and physically separates the epithelium from direct contact with much of the luminal microbiota (2). The majority of intestinal epithelial cells are absorptive enterocytes, but the epithelium also contains a number of more specialized cell types, including Paneth cells (in the small intestine only), goblet cells, hormone-secreting enteroendocrine cells, microfold (M) cells, and tuft cells (2, 5). Indeed, even these subtypes are too generalized to fully reflect the diversity of intestinal epithelial cells. Recent single-cell sequencing data identified two subtypes of tuft cells and subclassified enteroendocrine cells beyond the eight subclasses previously reported (6).

The gut epithelium is continuously renewed by *Lgr5^+^* stem cells located in the base of the intestinal crypts. Newly formed precursor cells differentiate as they migrate away from the crypt toward the villus tip in the small intestine or luminal surface in the large intestine, where they are expelled into the lumen approximately every 4–5 days. The exception to this is Paneth cells, which are long-lived and instead move toward the crypt base (2, 5). Each cell type plays critical and distinct roles in intestinal function. Mucus-secreting goblet cells are crucial for maintenance of the luminal mucus layer and increase in frequency moving distally along the intestine, peaking at a frequency of approximately 25% of total epithelial cells in the distal colon (2). Small intestinal Paneth cells produce antimicrobial peptides and also contribute to stem cell maintenance and function through the production of Wnt3, pro-epidermal growth factor, and Notch ligands (2). M cells overlie gut-associated lymphoid tissues and facilitate the transport of luminal antigens to lymphoid cells, while tuft cells coordinate type 2 immune responses to parasites (5, 7, 8). Much of intestinal epithelial research, including a portion of that presented herein, has focused on the use of colorectal cancer cell lines to elucidate gut epithelial function. However, due to the heterogeneity of the intestinal epithelium *in vivo*, observations made from cell lines, which are not representative of all gut epithelial cell types, may be misleading. Recent advances in three dimensional intestinal epithelial organoid cultures, which differentiate into the various epithelial cell subtypes seen *in vivo*, are improving our ability to more effectively characterize intestinal epithelial function, and many of these studies will be highlighted in this review (9).

The gut-associated lymphoid tissues, including Peyer’s patches and isolated lymphoid follicles, are likely the most well-recognized portion of the intestinal immune system. However, the entire gut is armed with a diverse repertoire of immune cells, which vary in location and frequency throughout the length of the intestine (2). The majority of these cells function in the lamina propria or within the epithelium of the intestinal mucosa. The epithelium predominantly hosts T cells, while the lamina propria is home to cells of both the adaptive and innate arms of the immune system, including T cells, B cells, innate lymphoid cells (ILCs), macrophages, dendritic cells, mast cells, and eosinophils (2). Immune cells may sense luminal antigens directly when the epithelial barrier is breached or by the extension of transepithelial dendrites, as has been observed in macrophages and dendritic cells. The intestinal epithelium is uniquely positioned and equipped with a cadre of pattern recognition receptors to sense luminal antigens and danger signals and relay this information to immune cells (2).

The intestinal epithelium faces the difficult challenge of permitting nutrient absorption and ion movement while maintaining an impermeable barrier to microorganisms and antigens in the gut lumen. The integrity of the intestinal mucosal barrier is critical for health; dysfunction of this barrier has been proposed to contribute to both intestinal and systemic disease, including inflammatory bowel disease (IBD) and multiple organ dysfunction syndrome (10, 11). Intestinal epithelial cells are linked by three types of specialized junctional complexes that attach adjacent cells and permit the selective paracellular movement of solutes and ions: desmosomes, adherens junctions, and tight junctions (10, 11). Desmosomes and adherens junctions predominantly serve as physical attachments between cells, while the more apically located tight junctions act as selective semipermeable barriers to intercellular spaces (12). Tight junctions are composed of four types of transmembrane proteins: junctional adhesion molecules, claudins, occludin, and tricellulin. Claudins are a family of proteins that are differentially expressed between tissues and exert different effects on paracellular permeability. Claudins critically regulate the selectivity of the epithelial barrier by forming charge- and size-specific channels between epithelial cells (12). The types of claudin proteins within tight junctions determine the permeability of these paracellular channels. For example, claudin-2 and claudin-6 have been shown to increase tight junction permeability. Intracellular zonula occludens proteins connect tight junction transmembrane proteins to cytoskeletal actin/myosin complexes, which facilitate opening of the tight junction under specific conditions (11, 12).

Cytokines and chemokines, soluble protein mediators critical for intercellular communication, support intestinal mucosal homeostasis but can also be key drivers of intestinal inflammation and inflammation-associated damage (1, 10, 13). For example, the genetic deletion of interleukin (IL)-10 or IL-2 precipitated spontaneous colitis in mice, suggesting that these cytokines are essential for colon homeostasis. However, a number of other cytokines, including IL-6, tumor necrosis factor (TNF), IL-18, IL-1β, and IL-17, are overexpressed in the inflamed intestine and have been implicated as contributors to intestinal damage (10). Despite these seemingly clear-cut observations, there is strong evidence that the traditional labels of pro- and anti-inflammatory are too simplistic and perhaps even deceiving when used to describe cytokine actions in the intestine. In support of this, clinical trials targeting cytokines thought to be predominantly pro-inflammatory in the intestine, such as IL-17, failed to induce remission in patients with IBD (10, 14). In addition, the literature contains conflicting and often equally convincing evidence for both pro- and anti-inflammatory actions of specific cytokines in the gut (10, 15). There are a number of potential explanations for these conflicting data, such as the timing of cytokine action, model system used, cytokine concentration, and the method of cytokine administration or removal (15–17). As such, cytokine actions should be interpreted on a situational basis to gain a more complete understanding of their diverse roles in health and disease.

**Figure 1:**
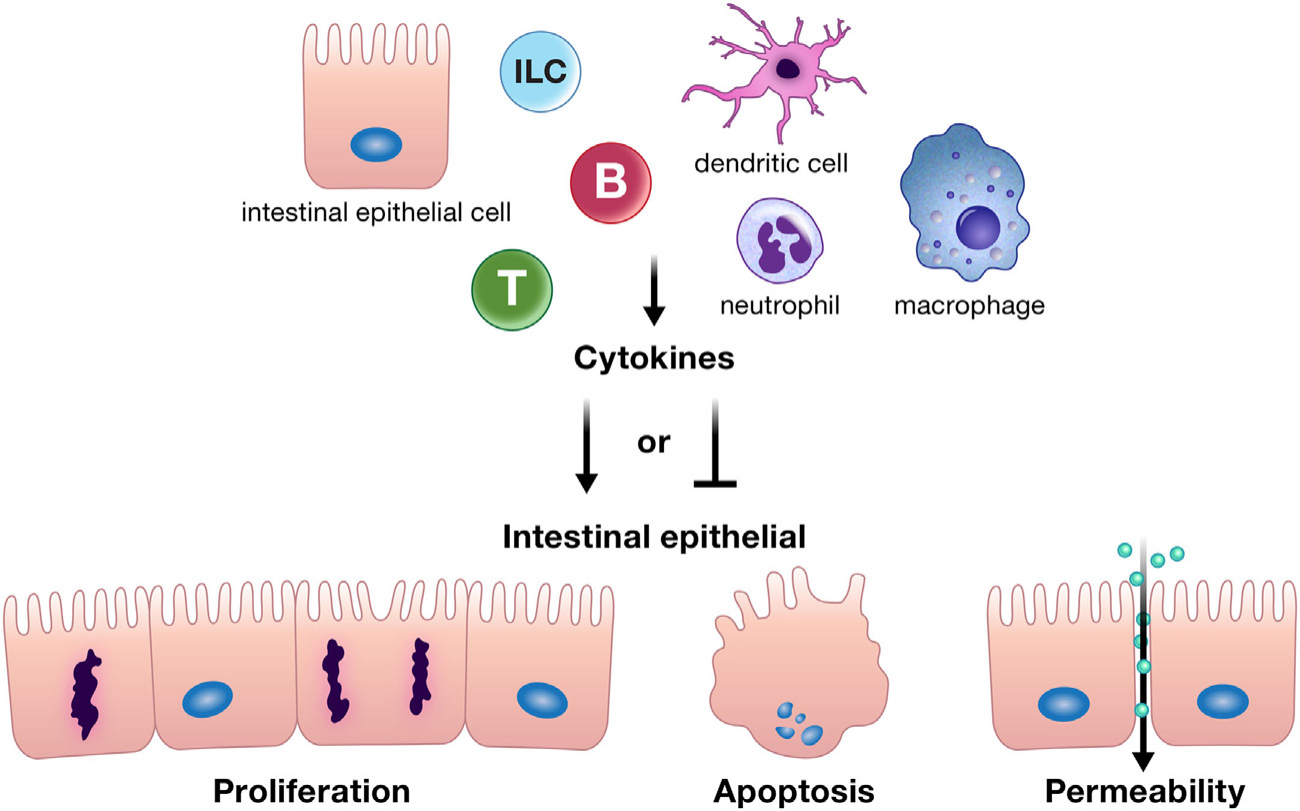
Cytokines can positively or negatively affect intestinal epithelial barrier integrity by driving or inhibiting critical epithelial cell functions such as proliferation, apoptosis, and appropriate epithelial barrier permeability. These cytokines can be derived from resident innate or adaptive immune cells, infiltrating inflammatory cells, or from intestinal epithelial cells themselves. Abbreviations: T, T cell; B, B cell; ILC, innate lymphoid cell.

Cytokines and chemokines can positively or negatively affect intestinal epithelial barrier integrity, and may be derived from resident innate or adaptive immune cells, infiltrating inflammatory cells, or from intestinal epithelial cells themselves (Figure 1) (10–12, 18–20). Intestinal epithelial proliferation and cell death can be induced or restricted by cytokines (21–23). Concordantly, various cytokines help heal the epithelial erosions and ulcerations characteristic of severe intestinal inflammation, while others exacerbate these lesions (10). Specific cytokines have also been shown to regulate opposing epithelial functions under different circumstances, for instance, proliferation or cell death (16, 22, 24–26). In addition, cytokines can directly alter intestinal epithelial permeability (27, 28). The permeability of epithelial tight junctions may be increased or decreased by cytokine modification of the expression or localization of their protein components (11, 12, 27, 29, 30). Cytokines can also drive phosphorylation of myosin light chains, resulting in contraction and opening of tight junctions (11). Chemokine production by the intestinal epithelium recruits immune cells to areas of inflammation; however, whether this means epithelial suicide or survival depends on the inflammatory insult. Recruited immune cells may be crucial for defense against a pathogen but can perpetuate inflammation in conditions such as IBD (31–34). Regardless of mechanism, cytokines and chemokines are critical players in the integrity of the intestinal epithelial barrier. The purpose of this review is to highlight recent advances in our understanding of how cytokines and chemokines, both those made by and acting on the intestinal epithelium, orchestrate many of the diverse functions of the intestinal epithelium and its interactions with immune cells in health and disease.

## Cytokine Actions on the Intestinal Epithelium

### Cytokine Stimulation of Intestinal Epithelial Proliferation

**Figure 2:**
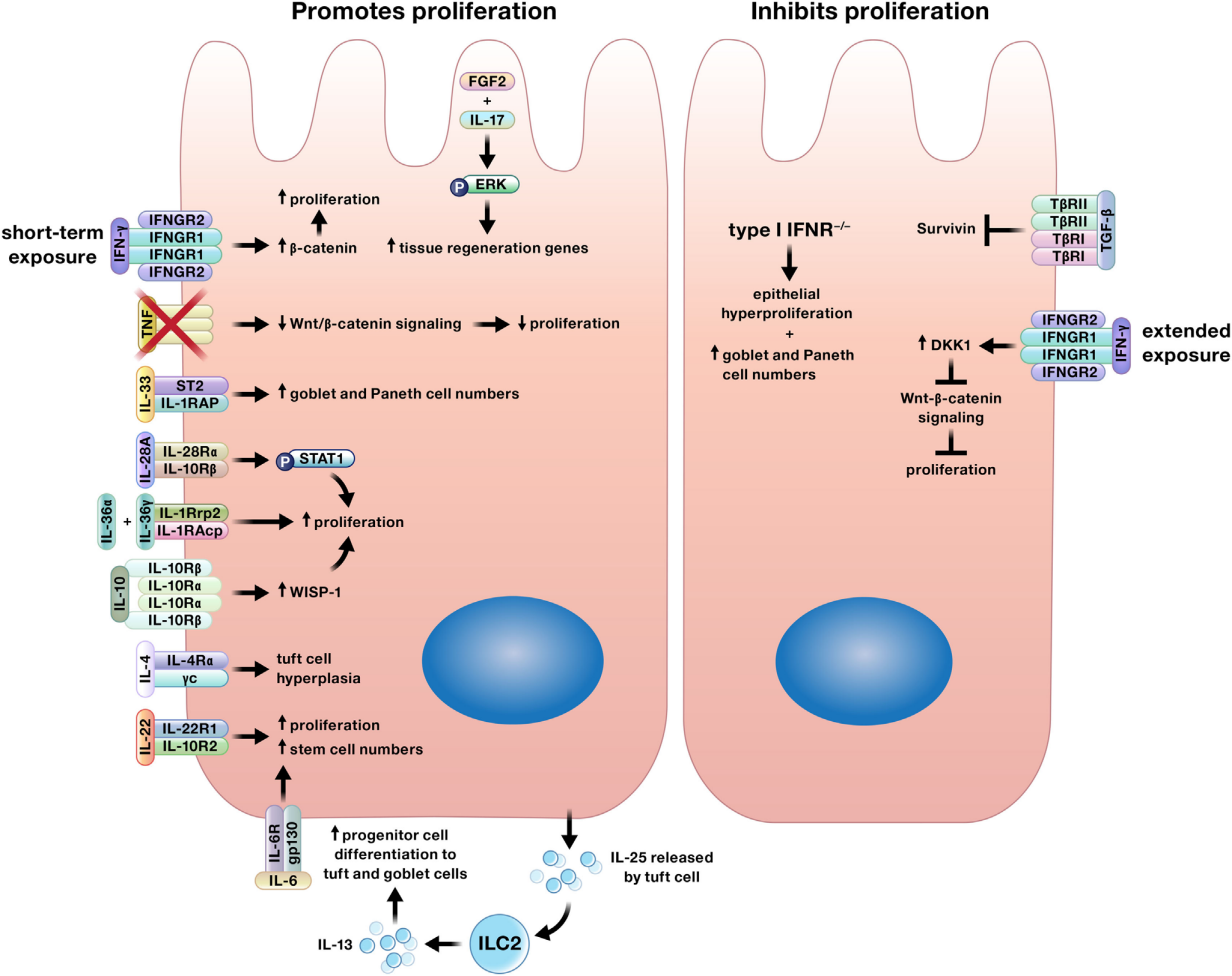
Cytokines may promote or inhibit proliferation of intestinal epithelial cells. Interferon (IFN)-γ may induce or limit intestinal epithelial proliferation based on duration of exposure. In addition, specific cytokines may only induce proliferation of certain epithelial subtypes. For example, interleukin (IL)-4 increases tuft cell numbers, IL-13 signaling supports increases in tuft and goblet cells, and IL-33 stimulates the expansion of goblet and Paneth cells.

Multiple cytokines regulate proliferation of the intestinal epithelium, a function that is crucial for both wound closure and replacing cells lost through homeostatic shedding (Figure 2) (7, 8, 16, 18, 35–50). Although generally thought to contribute to the pathology of IBD, recent studies have shown that TNF, IL-6, and IL-17 promote epithelial proliferation (14, 16, 18, 21, 44).

#### Tumor Necrosis Factor

In murine models of T cell activation and chronic chemically induced colitis, genetic ablation of either TNF or its receptor impaired Wnt/β-catenin signaling, resulting in reduced epithelial proliferation and delayed mucosal healing (16). This result may seem curious in light of the success of anti-TNF therapy in IBD patients; however, the authors offer an explanation for this perceived conflict by highlighting the mechanism of action of efficacious versus ineffective anti-TNF therapies. Therapeutic anti-TNF antibodies reduce inflammation in IBD patients by inducing apoptosis in inflammatory cells expressing membrane-bound TNF (51). By contrast, treatment with a soluble TNF receptor, which was ineffective in treating Crohn’s disease, binds soluble TNF, which the authors propose blocks the ability of TNF to promote mucosal healing (16, 52).

#### Interleukin-6

Interleukin-6 increased proliferation and stem cell numbers in an *in vitro* model of murine small intestinal epithelial organoids, and the crypt epithelial cells also expressed IL-6, suggesting an autocrine signaling mechanism. Interestingly, the IL-6 receptor was only present on the basal membrane of crypt Paneth cells, making it unclear how IL-6 may affect epithelial cells in segments of the intestine lacking Paneth cells, such as the colon (18). However, Paneth cell metaplasia can be found in various types of colitis, in which case this mechanism of IL-6-facilitated epithelial repair could play a role (53). Furthermore, Kuhn et al. demonstrated that the early inhibition of IL-6 in murine models of bacterial colitis and wounding by biopsy impaired colon wound healing by limiting epithelial proliferation. They also demonstrated by *in situ* hybridization that IL-6 mRNA transcripts were enriched within the mucosa surrounding sites of intestinal perforation in human patients, suggesting that this IL-6-driven mechanism of wound healing may also be important in humans. These findings suggest that while Paneth cells may be crucial for IL-6-induced epithelial proliferation in the small intestine, other mechanisms exist for IL-6 to drive epithelial repair in the colon (45).

#### Interleukin-17

Similarly, genetic ablation of IL-17 reduced intestinal epithelial cell proliferation and worsened dextran sulfate sodium (DSS)-induced murine colitis (44). Furthermore, IL-17 was shown to synergize with fibroblast growth factor 2 (FGF2) to promote intestinal healing in this study. FGF2 and IL-17 signaling synergistically activated ERK and induced genes related to tissue repair and regeneration in primary murine intestinal epithelial cells. The authors demonstrated that the mechanism of this synergy depended on Act1, an adaptor molecule that suppresses FGF2 signaling but is required for IL-17 signaling. When cells were co-stimulated with IL-17 and FGF2, Act1 was preferentially recruited to IL-17 receptors, preventing Act1-mediated suppression of FGF2 signaling (44). These findings may offer one explanation for the unexpected results of a clinical trial investigating the inhibition of the IL-17 receptor as a therapy for active Crohn’s disease, in which a disproportionate number of patients actually experienced worsening disease with treatment (14).

#### Interleukin-22

Interleukin-22 increased growth in both human and murine intestinal organoids, both by inducing proliferation of the epithelial cells and facilitating stem cell expansion (46). IL-22 was also shown to be crucial for stem cell maintenance *in vivo* in the small intestine in a murine model of methotrexate-induced intestinal damage (54). During *Citrobacter rodentium* infection, IL-22 production by CD4^+^ T cells was critical for colonic epithelial proliferation and resistance to infection-induced mucosal pathology (55).

#### Interleukin-36

Induction of IL-36 receptor signaling through any one of its ligands, IL-36α, IL-36β, or IL-36γ, induced proliferation of intestinal epithelial cells in *in vitro* organoid cultures, and mice with genetic deletion of the IL-36 receptor were more susceptible to chemically induced colitis, demonstrating higher disease activity, more severe colon pathology, greater bacterial translocation, and decreased survival. Furthermore, administration of a combination of IL-36α and IL-36γ accelerated wound healing in murine colons by increasing proliferation of epithelial cells adjacent to the experimental wounds (47).

#### Interleukin-28A

Similarly, IL-28A [also termed interferon (IFN) λ2] induced phosphorylation of signal transducer and activator of transcription 1 (STAT1) and proliferation in murine small and large intestinal epithelial organoid cultures (39). Mice with global knockout of the IL-28A receptor or intestinal epithelial cell-specific knockout of STAT1 developed more severe oxazolone and DSS-induced colitis, and the administration of IL-28A or genetic ablation of the IL-28A receptor in mice with induced colon wounds improved or delayed wound healing, respectively. The authors went further to link their murine models to human patients with IBD, demonstrating that both IBD patients and mice with colitis showed increased expression of the IL-28A receptor on the colon epithelium, as well as higher expression of IL-28A by cells within the lamina propria of the colon mucosa. Co-labeling of lamina propria cells in IBD patients identified dendritic cells as a major source of IL-28A (39).

#### Interleukin-10

A separate study also highlighted innate immune cells as a crucial cytokine source for mucosal healing. In a murine model of biopsy-induced colon injury, macrophage-derived IL-10 was crucial for optimal wound healing (48). IL-10 mRNA and protein were increased at wound sites within 1 day of wounding, and IL-10 induced epithelial proliferation by stimulating synthesis of Wnt1-inducible signaling protein-1. Interestingly, the absence of T and B cells in *Rag*1^−/−^ mice also used in this study did not impair wound closure, further highlighting macrophages as the primary source of IL-10 in this model and suggesting that adaptive immune cells do not play a crucial role in this mechanism of wound healing (48).

#### IL-13, IL-4, and IL-33 Support the Differentiation of Specialized Epithelial Cells

Expansion of tuft cells, a specialized taste-chemosensory subtype of the intestinal epithelium, can also be induced by innate immune cells. During helminth infection, IL-25 secreted by tuft cells activates type 2 ILCs to produce IL-13, which induces the differentiation of increased numbers of tuft and goblet cells from epithelial progenitor cells (7, 8). IL-4, which shares the common receptor subunit IL-4 receptor α with IL-13, can also induce tuft cell hyperplasia (49). Mahapatro et al. demonstrated that IL-33 also directly affected the differentiation of epithelial progenitor cells. The constitutive expression of IL-33 in the small intestine of mice increased goblet and Paneth cell numbers but did not promote the proliferation/differentiation of absorptive enterocytes. Challenge of IL-33^−/−^ mice with *Salmonella* Typhimurium demonstrated that IL-33 was critical for microbial defense, as mice lacking IL-33 had more severe intestinal damage and a greater *Salmonella* burden associated with decreased numbers of goblet and Paneth cells and reduced antimicrobial peptide production (50). Similarly, mice with genetic deletion of IL-33 or its receptor had decreased numbers of goblet cells and more severe colitis in a model of oxazolone-induced intestinal inflammation (17).

### Cytokine-Induced Proliferation and Carcinogenesis

In the absence of wound closure, cytokine-induced intestinal epithelial proliferation may prove to be more deleterious than healing. In fact, a number of studies have suggested that cytokines, including IL-17, IL-6, IL-22, TNF-α, IL-4, and IL-13, either alone or in combination, may promote carcinogenesis in intestinal epithelial cells (56–60). Wang et al. demonstrated that IL-17 receptor type A (IL-17RA) signaling promoted proliferation of transformed colon enterocytes. IL-17RA signaling also induced IL-6 expression, a cytokine previously associated with colitis-associated cancer development (56). The concurrent neutralization of either IL-6 and IL-22 or TNF-α and IL-17A inhibited NF-κB or STAT3 signaling, respectively, and reduced the mitogenic effects of these cytokines on human colorectal cancer cells (57). Multiple studies have also shown that IL-22 alone can promote colorectal cancer progression (58, 59).

Furthermore, both IL-4 and IL-13 may contribute to colon cancer progression. IL-4 and IL-13 increased the expression of NADPH oxidase 1 in human colon cancer cell lines, which led to the production of reactive oxygen species and cellular proliferation. When examined in resected tissues from patients with colon cancer, the authors found increased active NADPH oxidase 1 in the tumor tissue relative to the adjacent normal colon tissue, leading them to suggest that IL-4/IL-13-driven NADPH oxidase 1 expression may drive colon carcinogenesis (60).

### Cytokine Inhibition of Intestinal Epithelial Proliferation

In complement to the plethora of proliferation-inducing cytokines detailed earlier, a smaller number of cytokines limit intestinal epithelial proliferation (Figure 2) (24, 61–64).

#### Transforming Growth Factor-β (TGF-β)

Transforming growth factor-β suppressed expression of Survivin, a molecule critical for functional cell division in intestinal epithelial progenitor cells (61). Consistent with this finding, genetic disruption of TGF-β signaling in intestinal epithelial cells was sufficient for the development of invasive colon cancer in the face of chronic inflammation in mice (62).

#### Interferons

In a model of constitutive β-catenin signaling, Katlinskaya et al. demonstrated that type I IFNs limit intestinal epithelial proliferation (63). Concordantly, Tschurtschenthaler et al. characterized mice with intestinal epithelial-specific genetic deletion of the type I IFN receptor as having increased numbers of small intestinal goblet and Paneth cells, epithelial hyperproliferation, and increased tumor burden following tumor induction with azoxymethane and DSS (64). Remarkably, the authors were able to eliminate the epithelial hyperproliferation and increase in tumors by cohousing the type I IFN receptor knockout mice with wild-type mice, demonstrating that these knockout-induced phenotypes were dependent on the gut microbiota (64).

The effects of the type II IFN, IFN-γ, on the intestinal epithelium vary with length of exposure. The short-term incubation of the intestinal epithelial cell line T84 with IFN-γ activated β-catenin signaling and induced proliferation of the T84 cells, peaking at 24 h. However, extended exposure of the T84 cells to IFN-γ induced expression of DKK1, which inhibited Wnt-β-catenin signaling and reduced proliferation. Interestingly, the addition of both TNF-α and IFN-γ enhanced these effects (24).

### Damage Control: Cytokine Regulation of Apoptosis

**Figure 3:**
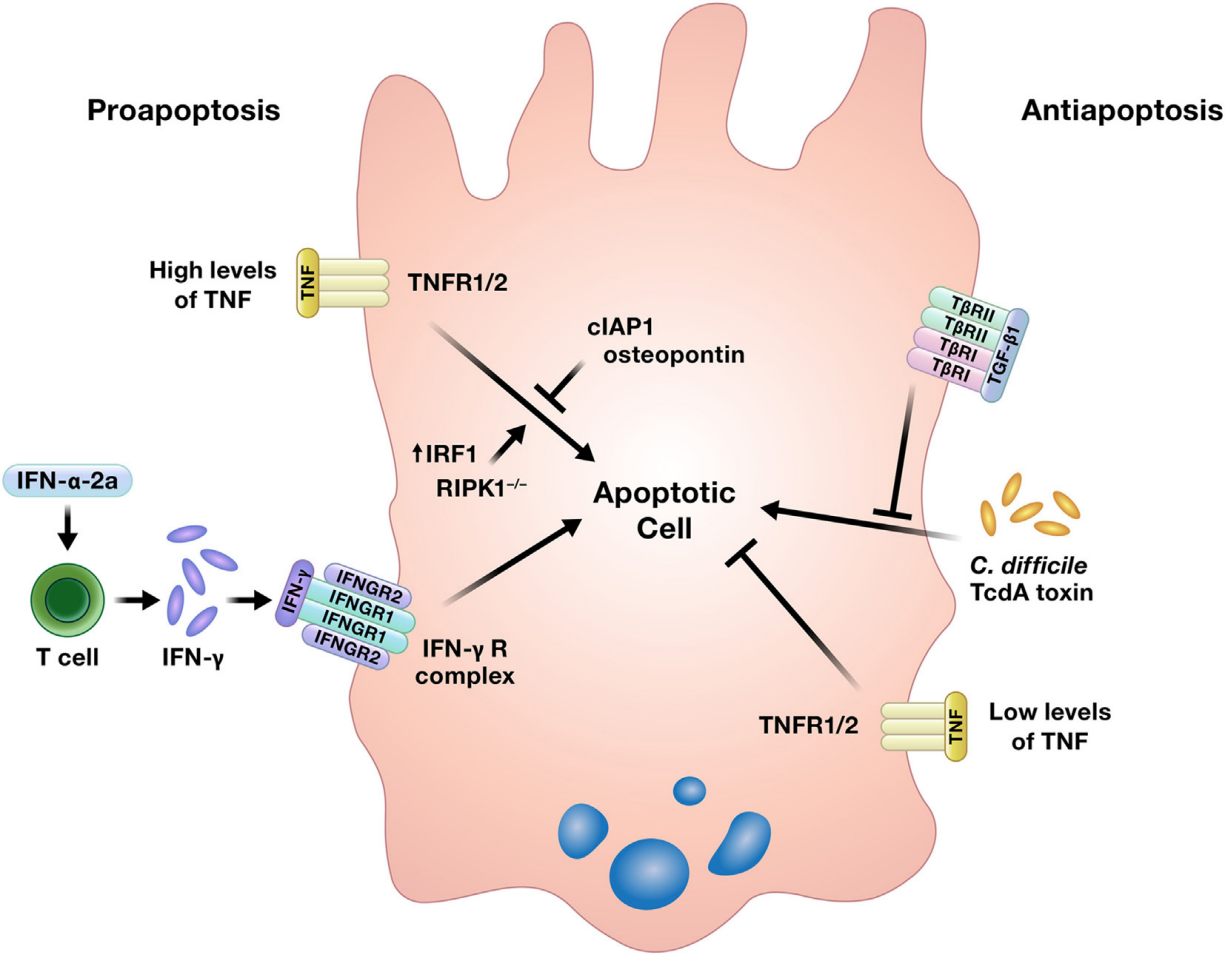
Cytokines can induce or prevent apoptosis in intestinal epithelial cells. TNF has been shown to either promote or inhibit intestinal epithelial cell apoptosis under different conditions. Abbreviations: IAP, inhibitor of apoptosis protein; IRF1, interferon regulatory factor 1; RIPK1, receptor interacting protein kinase 1; TNF, tumor necrosis factor.

While well-regulated apoptosis is essential for the homeostatic shedding of enterocytes, any perturbations to this process could quickly compromise the intestinal epithelial barrier. Indeed, increased apoptosis has been detected in the intestinal epithelium of IBD patients, although it is unclear if this is an initiating event in the disease, an effect of inflammation, or some combination of both (5). Increased intestinal epithelial apoptosis is also a consistent feature in critically ill humans and animal models of critical illness, such as sepsis. This increase in apoptosis contributes to intestinal epithelial barrier compromise in critical illness, which has been implicated as a critical driver of multiple organ dysfunction syndrome (11). Cytokines can induce or inhibit intestinal epithelial apoptosis (Figure 3) (16, 22–26, 65–67).

#### Interferons

Interferons have been shown to induce apoptosis of intestinal epithelial cells. Using human colon explant cultures, Jarry et al. demonstrated that administration of IFN-α-2a rapidly induced IFN-γ production by lamina propria resident T cells and IFN-γ-dependent epithelial apoptosis, a direct effect of IFN-γ on the intestinal epithelium that has been reported previously (24, 65, 66). Katlinskaya et al. also demonstrated a role for type I IFN in promoting apoptosis of the intestinal epithelium in a model of constitutive β-catenin signaling (63).

#### Tumor Necrosis Factor

In contrast to its ability to promote intestinal epithelial proliferation, one of the most well-characterized actions of TNF in the intestine is its ability to induce epithelial cell death. Injection of mice with TNF results in increased apoptosis of both small and large intestinal epithelial cells within 6 h, with a concentration of apoptotic cells in the intestinal crypts. Exposure of intestinal epithelial organoids derived from mice with genetic deletion of TNF receptors 1 and 2 revealed that while both receptors participated in TNF-mediated epithelial apoptosis, TNF receptor 1 signaling was predominantly involved. The authors further demonstrated that TNF-induced intestinal epithelial apoptosis is regulated by the inhibitor of apoptosis protein cIAP1. Inhibition of cIAP1 by second mitochondrial activator of caspases-mimetic compounds, tumor necrosis factor-related weak inducer of apoptosis (TWEAK), or genetic deletion sensitized mice to TNF-induced intestinal epithelial apoptosis (22). A separate *in vitro* study using cancerous and non-cancerous colon epithelial cell lines demonstrated that osteopontin reduced TNF-induced apoptosis, while the overexpression of IFN regulatory factor 1 increased TNF-mediated apoptosis (25). TNF was also implicated as contributing to the pathogenesis of intestinal inflammation in mice with conditional knockout of receptor interacting protein kinase 1 (RIPK1). Full RIPK1 knockout mice die perinatally, but the conditional RIPK1 knockout in intestinal epithelial cells in mice used in this study resulted in intestinal inflammation and early death associated with epithelial cell apoptosis. However, this phenotype was rescued by a deficiency in TNF receptor 1, and the lack of RIPK1 in *in vitro* cultured intestinal epithelial organoids sensitized the cultures to TNF-induced apoptosis (26).

In lieu of apoptosis, under certain circumstances, cells may undergo the pro-inflammatory process of regulated necrosis termed necroptosis (68). In addition to its ability to drive apoptosis, TNF can also initiate necroptosis of intestinal epithelial cells under specific conditions. In a model of conditional knockout of caspase 8 in intestinal epithelial cells, Günther et al. demonstrated that necroptosis in gut epithelial cells was triggered by TNF-α produced by other cells upon bacterial lipopolysaccharide (LPS) stimulation, not direct LPS-induced toll-like receptor 4 (TLR4) signaling in the epithelium. By contrast, gut epithelial necroptosis due to TLR3 ligation in the same model was cytokine-independent and directly initiated by TLR3 signaling (69).

In light of the strong evidence for a pro-apoptotic function of TNF in the gut, Bradford et al. curiously demonstrated an anti-apoptotic effect of TNF in the intestinal epithelium. In the murine model of T cell activation induced by anti-CD3 antibody injection used in this study, intestinal epithelial apoptosis is expected both acutely at the villus tips and later in the crypts around 24 h post-injection. Interestingly, and perhaps counterintuitive to the evidence presented herein thus far, administration of anti-CD3 antibody in TNF^−/−^ mice resulted in a sevenfold increase in crypt epithelial apoptosis, suggesting that TNF works to limit epithelial apoptosis in this model (16). Other studies have also characterized an anti-apoptotic role for TNF in the intestinal epithelium, and the authors suggest that the level of TNF may determine whether it acts to promote or prevent apoptosis, with higher levels of TNF proposed to be pro-apoptotic (16, 67).

#### Transforming Growth Factor-β1

Transforming Growth Factor-β1 can also inhibit intestinal epithelial cell death. TGF-β1 reduced apoptosis and prevented necrosis in rat jejunal crypt epithelial cells exposed to the TcdA toxin of *Clostridium difficile* (23).

### Cytokine Reinforcement of Intestinal Epithelial Barrier Integrity

**Figure 4:**
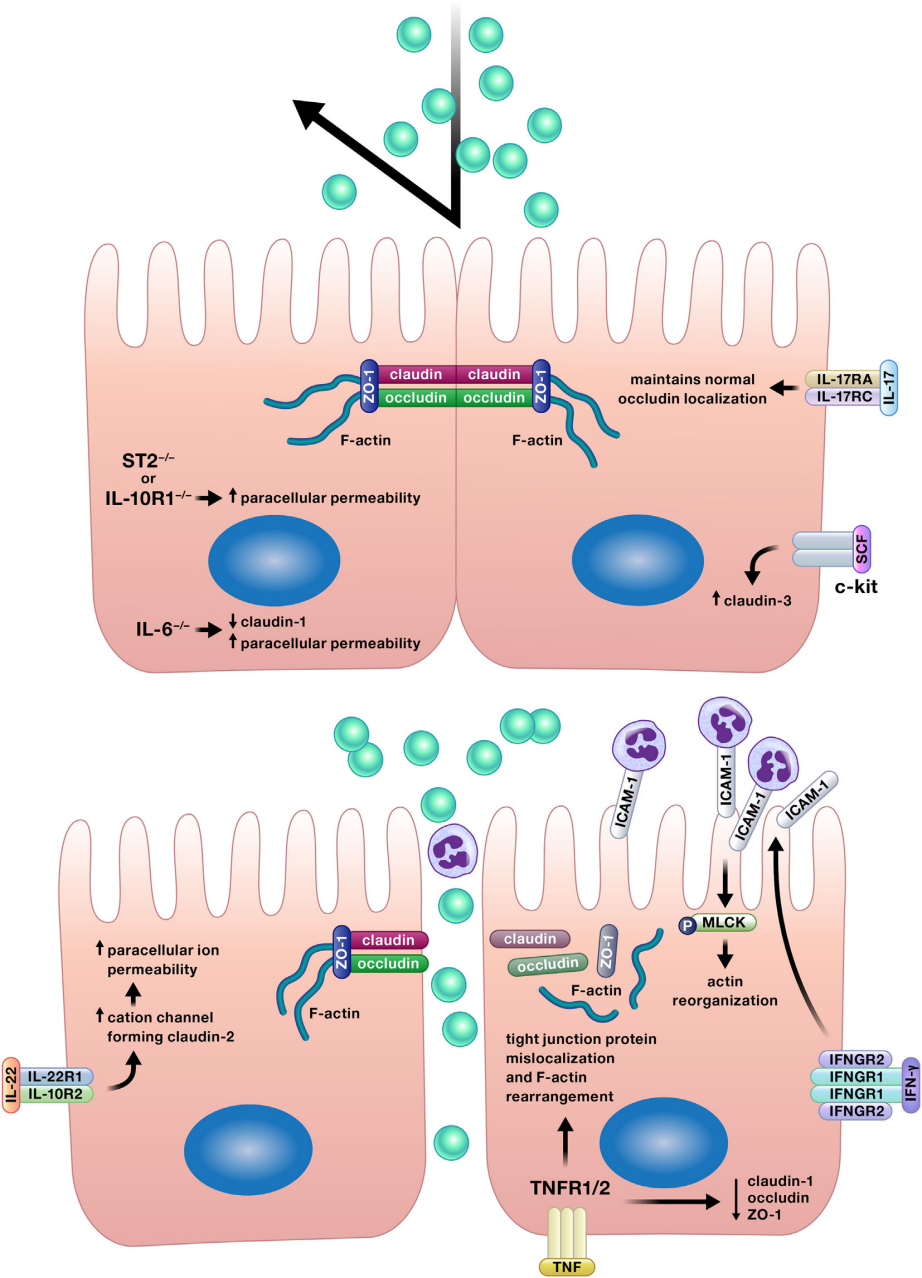
Appropriate permeability of the intestinal epithelium maintains balance between nutrient absorption and pathogen exclusion. Cytokines may reinforce or impair the intestinal barrier by altering permeability of the epithelium. Epithelial tight junction permeability may be increased or decreased by cytokine modification of the expression or localization of tight junction protein components, such as various claudins, occludin, or zonula occludens protein-1 (ZO-1). Cytokines can also drive phosphorylation of myosin light chains, resulting in contraction and opening of tight junctions. Interferon (IFN)-γ increases intercellular adhesion molecule-1 (ICAM-1) expression, and subsequently, ICAM-1-mediated adherence of neutrophils to gut epithelial apical membranes. Neutrophil ligation of ICAM-1 drives the phosphorylation of myosin light-chain kinase (MLCK), resulting in actin reorganization leading to increased paracellular permeability and neutrophil transepithelial migration.

Appropriate permeability of the intestinal epithelium is crucial for the balance between nutrient absorption and pathogen exclusion, and a number of cytokines positively affect this epithelial function (Figure 4) (12, 17, 27, 42, 70–72).

#### Interleukin-17

Inhibition of IL-17 receptor A by antibody neutralization worsened disease in the multidrug resistance-1a-ablated (*Abcb1a^−/−^*) murine model of colitis and was associated with increased epithelial permeability as detected by increased serum concentrations of soluble CD14 and LPS binding protein and increased plasma concentrations of orally administered sucralose, lactulose, and mannitol (70). Lee et al. also demonstrated that a loss of IL-17 signaling increased intestinal epithelial permeability by showing increased amounts of orally administered fluorescein isothiocyanate (FITC)–dextran in the serum of mice with both chemically induced and T cell transfer-induced colitis in which IL-17 was removed by antibody neutralization or genetic deletion (27). The authors attributed the increase in gut epithelial permeability in the absence of IL-17 to disruptions in the structure of tight junctions, junctional complexes which are critical to the selectivity inherent in appropriate gut barrier permeability. The absence of IL-17 resulted in the intracellular mislocalization of the tight junction complex protein occludin and a loss of co-localization of occludin with F-actin. To provide more support for this mechanism, the authors applied TNF-α, a cytokine previously reported to disrupt tight junctions and increase epithelial barrier permeability, to cultured Caco-2 cells with or without co-stimulation with IL-17A (27, 28). Consistent with their observations *in vivo*, TNF-α altered the intracellular localization of occludin; however, co-stimulation with IL-17A reduced the TNF-induced occludin mislocalization (27). Along with the previously described ability of IL-17 to induce intestinal epithelial regeneration, the ability of IL-17 to reinforce the intestinal epithelial barrier offers an additional potential explanation for the worsening of Crohn’s disease observed in clinical trial patients treated with an antibody to inhibit IL-17 receptor signaling (14).

#### Interleukin-10

Multiple studies have shown the positive effects of IL-10 signaling in the gut epithelium for maintenance of appropriate epithelial permeability (42, 73, 74). Stimulation of T84 cell monolayers with IL-10 restored transepithelial electrical resistance disrupted by compromise of the monolayers by incubation with IFN-γ. In addition, knockdown of the IL-10 receptor 1 in human intestinal epithelial cell lines impaired barrier formation as assessed by transepithelial electrical resistance and increased paracellular flux (42). These changes suggest alterations in the function of intercellular tight junctions owing to the lack of IL-10 signaling; however, this potential mechanism was not explored in this study. In the same study, mice with intestinal epithelial cell-specific knockout of the IL-10 receptor 1 developed more severe chemically induced colitis with increased epithelial permeability to FITC–dextran (42). The authors concluded that the more severe colitis in these mice was driven by increased barrier permeability due to a lack of IL-10 signaling in epithelial cells. However, as previously discussed, IL-10 can induce proliferation in intestinal epithelial cells (48). As such, the inhibition of IL-10-induced epithelial restitution could have also contributed to the more severe colitis demonstrated in mice lacking intestinal epithelial expression of the IL-10 receptor 1 in this study.

In a separate study, Zheng et al. demonstrated how a cytokine, in this case IL-10, can interact with the intestinal microbiota to regulate epithelial function (73). Butyrate, a short chain fatty acid made by the intestinal microbiota *in vivo*, induced the expression of both IL-10 receptor α subunit mRNA and protein in T84 and Caco-2 cells. Treatment of T84 cells with butyrate and IL-10 increased epithelial barrier integrity more than butyrate alone as determined by increased transepithelial electrical resistance. Based on the increased expression of the IL-10 receptor α subunit in the epithelial cells due to butyrate treatment, the mechanism for this increase in barrier integrity owing to butyrate and IL-10 could be hypothesized to be an increase in IL-10 signaling due to increased IL-10 receptor expression. However, the authors did not compare these data with the transepithelial electrical resistance induced by IL-10 in the absence of butyrate. As a result, it is unclear from these data whether butyrate and IL-10 synergistically increase transepithelial electrical resistance in intestinal epithelial cells, or if the level reported in this study could have been induced by IL-10 alone. The authors went further to demonstrate that butyrate reduced both the mRNA and protein expression of the pro-permeability tight junction protein claudin-2 in T84 cells in an IL-10 receptor α-dependent manner, providing a potential mechanism for the observed increases in epithelial barrier integrity in the presence of butyrate (73). Interestingly, reductions in butyrate-producing bacteria have been reported in the microbiota of ulcerative colitis patients, suggesting a potential mechanism of epithelial barrier compromise due to dysbiosis as a contributing factor in this disease (75).

A study by Lorén et al. demonstrated how IL-10 can increase the effectiveness of other therapies (74). Previous work by this group correlated low IL-10 mRNA levels with poor glucocorticoid response in active Crohn’s disease. In a later study, the authors discovered a possible mechanism for this observation, as treatment with a combination of IL-10 and glucocorticoids, but neither treatment alone, restored the transepithelial electrical resistance of Caco-2 cell monolayers following their disruption with TNF-α (74).

#### Interleukin-6

A study by Kuhn et al. provided more evidence for the crucial relationship between the microbiota, immune system, and intestinal epithelial barrier (71). Bacteria in the order Bacteroidales were sufficient to induce localization of intraepithelial lymphocytes in the colons of mice, and these cells were an important source of IL-6. IL-6 supported epithelial barrier function, as IL-6^−/−^ mice displayed reduced expression of the tight junction protein claudin-1, a thinner mucus gel layer, and augmented paracellular permeability, all defects which were resolved by the transfer of IL-6^+/+^ intraepithelial lymphocytes to affected mice (71).

#### Stem Cell Factor

C-kit signaling has also been shown to promote intestinal epithelial barrier integrity through the regulation of a tight junction protein. The overexpression of c-kit or administration of its ligand stem cell factor increased expression of the tight junction protein claudin-3 in colorectal cancer cells *in vitro*, and decreased claudin-3 expression was observed in the colon epithelium of mice lacking functional c-kit (72).

#### Interleukin-33

Rectal biopsies from adult and pediatric patients with ulcerative colitis have increased IL-33 expression relative to specimens lacking inflammation (17). To determine if this implicates IL-33 as a contributor to inflammation or an anti-inflammatory response in these patients, Waddell et al. investigated the role of IL-33 in chemically induced colitis in mice (17). Mice with genetic deletion of ST2, the receptor for IL-33, had decreased colon transepithelial electrical resistance and increased permeability to FITC–dextran, suggesting that IL-33 promotes colon epithelial barrier function. In support of these data, genetic deletion of either ST2 or IL-33 precipitated more severe chemically induced colitis in these mice (17). However, the authors did not fully characterize the mechanism by which IL-33 promoted epithelial barrier integrity in these studies. The authors reported that intestinal epithelial proliferation and apoptosis were unaffected by the absence of IL-33 or ST2 in this model of colitis, but that goblet cell numbers and *Muc2* expression were decreased in these mice. This suggests that alterations in the mucus layer could have influenced epithelial barrier permeability in these mice, but the mucus layer itself was not evaluated. In addition, potential effects of IL-33 on interepithelial junctional complexes were not assessed; however, the authors did demonstrate that IL-33-induced augmentation of transepithelial electrical resistance in T84 cell monolayers was dependent on ERK1/2 signaling (17). This is particularly curious in light of a recent paper that reported reduced transepithelial electrical resistance and claudin-1 expression induced by IL-33-stimulated ERK signaling in human keratinocytes (76). This discrepancy could be explained by the different cell types investigated; however, conflicting roles for IL-33 in intestinal inflammation have been reported. Other investigators have demonstrated exacerbation of multiple models of murine colitis and decreased intestinal epithelial barrier integrity due to the administration of IL-33 (77, 78). Waddell et al. suggest that these inconsistencies could be due to differences in IL-33 concentrations among studies or the differing characteristics of inflammation in each colitis model, two reasonable explanations that warrant further investigation (17). In support of the data reported by Waddell et al., Sattler et al. demonstrated the induction of protective IL-10-producing regulatory B cells by IL-33 (78). The administration of IL-33 accelerated spontaneous colitis in IL-10-deficient mice but did not induce intestinal inflammation in wild-type mice. In addition, the transfer of IL-33-induced, IL-10-producing regulatory B cells to IL-10-deficient mice reduced colitis severity and delayed disease onset (78). As previously discussed, IL-10 promotes epithelial barrier integrity (42, 73). As such, reduced IL-10 production owing to genetic ablation of IL-33 signaling is a potential mechanism for the increased intestinal epithelial permeability observed by Waddell et al. (17, 42, 73).

### Falling Through the Cracks: Cytokine Promotion of Intestinal Epithelial Permeability

In contrast to the barrier reinforcing properties of the cytokines described earlier, a handful of cytokines can also disrupt the intestinal epithelium and promote barrier permeability (Figure 4) (29, 30, 79, 80).

#### Tumor Necrosis Factor

Various effects of TNF-α on the intestinal epithelium discussed herein could disrupt the epithelial barrier; however, TNF-α stimulation of intestinal epithelial cells has also been specifically demonstrated to decrease the protein expression of the tight junction proteins claudin-1, occludin, and zonula occludens protein-1 (ZO-1), as well as to induce cytoskeletal F-actin rearrangement and the mislocalization of occludin and ZO-1 (29, 30). Multiple studies have identified mechanisms to reduce TNF-α-induced epithelial barrier compromise, including the overexpression of anterior gradient protein 2 homolog, rebeccamycin treatment, and the stimulation of muscarinic cholinoceptor-mediated signaling (29, 30, 81).

#### Interleukin-22

Interleukin-22 also increases gut epithelial permeability *via* manipulation of tight junction protein expression. IL-22 stimulation of Caco-2 cells *in vitro* and murine colon epithelial cells *in vivo* increased the expression of the tight junction protein claudin-2, which forms cation channels. Caco-2 monolayers treated with IL-22 displayed decreased transepithelial electrical resistance, indicating increased paracellular ion permeability, but no change in movement of uncharged macromolecules across the monolayers was observed (79).

#### Interferon-γ

The increase in intestinal epithelial permeability induced by IFN-γ described by Sumagin et al. provides an elegant example of the intricate relationships between cytokines, the epithelium, and immune cells (80). Using the T84 intestinal epithelial cell line for an *in vitro* model of transepithelial migration of neutrophils, the authors demonstrated that IFN-γ induced expression of the intercellular adhesion molecule-1 (ICAM-1) on the apical membrane of T84 cells and increased the number of neutrophils adherent to the apical epithelial membranes *via* ICAM-1 post-migration. The ligation of ICAM-1 by neutrophils resulted in the phosphorylation of myosin light-chain kinase and a subsequent increase in epithelial permeability characterized by actin cytoskeletal reorganization, paracellular FITC–dextran flux, and a decrease in transepithelial electrical resistance. Notably in this model, this increase in epithelial permeability facilitated neutrophil transepithelial migration (80).

### Additional Cytokine Effects on Intestinal Epithelial Function

In addition to those detailed earlier, cytokines modulate a wide array of other intestinal epithelial functions. While endogenous type III IFN produced by intestinal epithelial cells does not restrict human rotavirus replication due to viral antagonism of the type III IFN response, treatment of human rotavirus-infected small intestinal organoid cultures with exogenous type I IFN, and to a lesser extent exogenous type III IFN, limits rotaviral replication (82). However, other studies in mice have found that IFN-λ, a type III IFN, is more effective than type I IFNs in limiting viral replication in the intestinal epithelium in models of reovirus and rotavirus infection (83, 84).

In a somewhat unexpected role, IL-22 production by neutrophils in chemically induced murine colitis induced the expression of antimicrobial peptides by the colon epithelium and protected the epithelium from chemically induced damage (85). Epithelial signaling of the IL-17 receptor regulates colonization of the murine intestine with segmented filamentous bacteria through the epithelial expression of the apical NADPH oxidase *Nox1, polymeric immunoglobulin receptor* (*Pigr*), and α-defensins (86). In addition to the functions previously discussed, TNF stimulation of the intestinal epithelium has also been shown to reduce expression of the ${\text{Cl}}^{-}/{\text{HCO}}_{\text{3}}$ exchanging solute carrier family 26 member 3, which may represent a therapeutic target in IBD-associated diarrhea (87). TNF also augmented receptor activator of NF-κB ligand-induced M cell differentiation (88).

## Talking Back: Intestinal Epithelial-Derived Cytokines and Chemokines

### Pro- and Anti-Inflammatory Functions of Intestinal Epithelial-Derived Cytokines

The intestinal epithelium is not simply beholden to respond to immune cell-derived cytokines but is a rich source of cytokines and chemokines, which may ameliorate or promote inflammation. The colonic epithelium was found to be a larger source of trefoil factor 2 (TFF2) than colon leukocytes, and TFF2 was protective in both acute and chronic models of DSS-induced colitis (19). In models of helminth infection, production of IL-25 by intestinal epithelial tuft cells regulated the helminth-induced type 2 immune response and facilitated worm expulsion (7, 49).

Intestinal epithelial cells also produce the anti-inflammatory cytokine IL-10, which likely contributes to tolerance to commensal bacteria. TLR4 ligation induced intestinal epithelial cell expression of IL-10, and this expression was enhanced by co-culture with macrophages (20). As previously discussed, IL-10 has been shown to stimulate intestinal epithelial cell proliferation and reinforce the integrity of the epithelial barrier (42, 48, 73). Thus, the microbiota may contribute to intestinal epithelial integrity through epithelial TLR4 ligation and the subsequent autocrine action of epithelial-derived IL-10. If so, IL-10 would not be the only cytokine with an autocrine mechanism for promoting epithelial homeostasis. IL-6 production by the intestinal epithelium has also been detected, which was shown to act in an autocrine manner to regulate crypt homeostasis (18).

In contrast to these anti-inflammatory and homeostatic effects, intestinal epithelial products may also promote inflammation. The accumulation of visceral fat has been associated with chronic intestinal inflammation, and in support of this, coculture of intestinal epithelial cells with differentiated adipocytes induced epithelial expression of TNF and matrix metalloproteinase-9 (89). IL-1α release by necrotic intestinal epithelial cells in a murine model of chemically induced colitis induced cytokine production by mesenchymal cells and reactivated colon inflammation post-recovery when delivered *via* enema (90). The findings of Bersudsky et al. support these data, as genetic ablation of IL-1α ameliorated murine DSS-induced colitis (91).

Intestinal epithelial cells also secrete IL-33; however, there is conflicting evidence in the literature regarding its role in both IBD [reviewed by Griesenauer et al. (40)] and intestinal carcinogenesis. IL-33 expression was found to be increased in epithelial cells of both murine and human intestinal tumors, and IL-33 promoted tumor development in *Apc*^Min/+^ mice (92, 93). Similarly, the expression of IL-33 by intestinal epithelial cells was increased in the murine azoxymethane/DSS model of colon cancer, and the authors went further to demonstrate that the epithelial expression of IL-33 was driven by epidermal growth factor (94). By contrast, knockdown of the IL-33 receptor, ST2, in colon cancer cells from mice enhanced tumor growth, suggesting a potential antitumorigenic role for IL-33 (95).

### Calling in the Troops: Intestinal Epithelial Chemokine Production

Intestinal epithelial-derived chemokines can contribute to both cellular defense and pathology. *Listeria monocytogenes* infection of an intestinal epithelial cell line induced expression of the chemokines IL-8, CCL1, and CCL20. Consistent with the epithelial invasiveness of *L. monocytogenes*, the high levels of CCL20 and IL-8 were likely induced by intracellular TLR10 signaling, the knockdown of which reduced chemokine levels more than silencing of TLR1 or TLR2 (31). IL-8, CCL1, and CCL20 are responsible for neutrophil, Th2 and regulatory T cell, and Th17 and dendritic cell trafficking, respectively, and would promote the infiltration of these cell types in the infected mucosa (96). Interestingly, a separate study identified a non-chemotactic role for IL-8 in the intestine. Apically secreted intestinal epithelial cell-derived IL-8 in response to TLR2 and TLR5 ligation was shown to act in an autocrine manner to promote gene expression related to cellular differentiation (97).

Chemokines likely play a critical role in the perpetuation of intestinal inflammation in IBD patients. Dent et al. reported that cocultured eosinophils and intestinal epithelial cells synergized to increase neutrophil chemotactic activity and CXCL5 production; however, the authors did not quantify the individual contributions of each cell type to this increase (33). As evidence of activated eosinophils has been detected in acute flares of IBD, this could contribute to excessive neutrophil recruitment to the intestine and increased tissue damage in active IBD (33). Production of the cytokine IL-34 is increased in the intestine of patients with active IBD, and Franzè et al. demonstrated that production of the chemokine CCL20 was associated with IL-34 signaling in both the DLD-1 colon epithelial cell line and in mucosal explants from IBD patients (34). CCL20 production could fuel the inflammatory response in active IBD patients through the recruitment of Th17 and dendritic cells. However, the potential consequences of increased CCL20 production are not so clear-cut. In fact, these cells could aid in restitution of the epithelial barrier in IBD patients. As noted previously, IL-17 can increase intestinal epithelial cell proliferation and reduce barrier permeability, and dendritic cells are a critical source of IL-28A in the gut, another cytokine shown to induce intestinal epithelial proliferation (27, 39, 44, 70). Conversely, this hypothesized cytokine-induced proliferation could be too much of a good thing. IL-17 has been shown to both induce the proliferation of transformed enterocytes and stimulate IL-6 production, a cytokine implicated in colitis-associated carcinogenesis (56). The neutrophil chemokine CXCL1 has also been shown to promote carcinogenesis. The upregulation of CXCL1 by colon tumor epithelium was dependent on hypoxia-inducible factor 2α and contributed to colon carcinogenesis through neutrophil recruitment (32).

### Intestinal Epithelial Responses to Pathogens and Commensals

**Figure 5:**
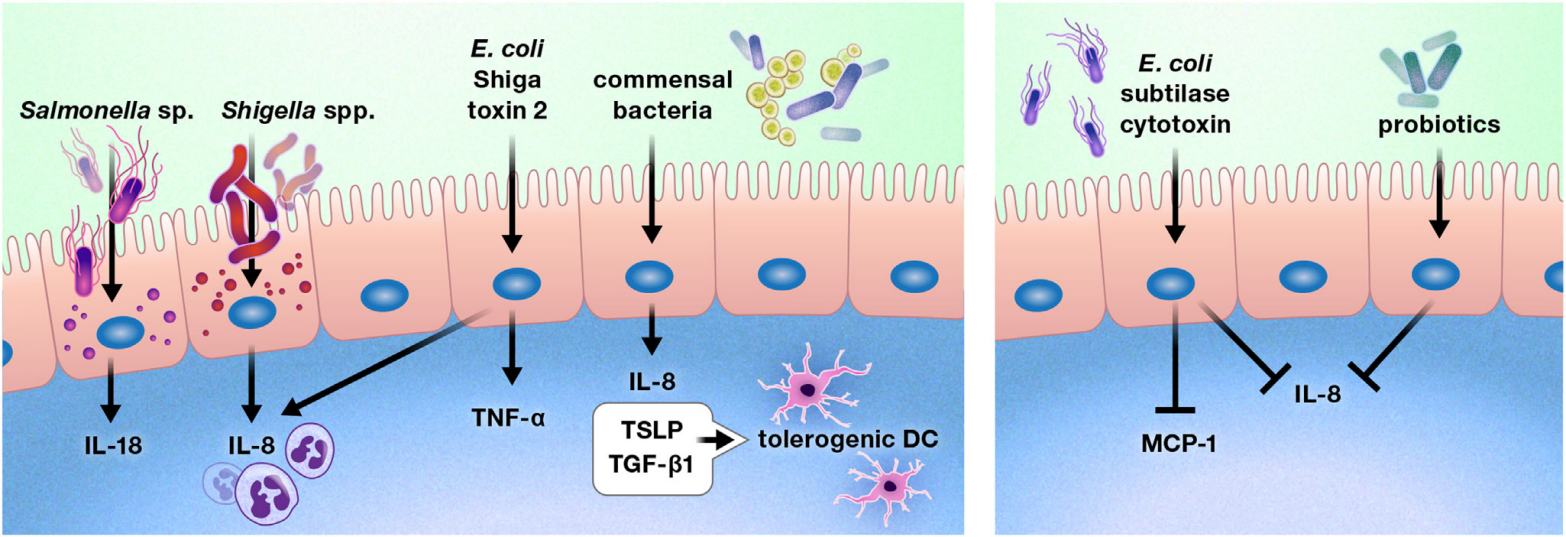
Pathogens, commensal bacteria, and probiotics can increase or diminish the production of cytokines and chemokines by the intestinal epithelium. These interactions may promote or deter immune cell infiltration of the gut, such as by increasing or reducing the production of chemokines, including interleukin (IL)-8 and monocyte chemoattractant protein-1 (MCP-1). In some cases, bacterial interactions with the gut epithelium may instruct the intestinal immune system. For example, intestinal epithelial cells produce thymic stromal lymphopoietin (TSLP) and transforming growth factor-β (TGF-β) 1 in response to commensal bacteria, inducing a tolerogenic phenotype in dendritic cells.

The intestinal epithelium is uniquely located to be the ideal first line of defense or communication with intraluminal bacteria and viruses. A number of bacteria alter cytokine production by the gut epithelium (Figure 5) (98–103). Exposure of the colon epithelial cell line HCT-8 to Shiga toxin 2 produced by Shiga-toxigenic *Escherichia coli* increased protein expression of IL-8 and TNF-α. However, HCT-8 exposure to subtilase cytoxin produced by the same bacterium decreased protein expression of IL-8 and monocyte chemoattractant protein-1 relative to unstimulated control cells, suggesting that these bacteria may use specific toxin production to differentially modulate host defenses (98). Infection of Caco-2 monolayers with *Shigella flexneri* 2a or *Shigella dysenteriae* 1 induced IL-8 secretion, which was predominantly released from the basolateral aspect of the epithelial cells, and *Salmonella enterica* serovar Typhimurium activated non-canonical inflammasome activity in murine and human intestinal epithelial cells, facilitating IL-18 secretion and bacterial clearance (99, 100).

In contrast to these predominantly pro-inflammatory responses, stimulation of Caco-2 cells with commensal bacteria increased thymic stromal lymphopoietin (TSLP), IL-8, and TGF-β1 secretion, which resulted in the promotion of a tolerogenic dendritic cell phenotype by TSLP and TGF-β1 (101). In addition, probiotic bacterial strains have been shown to reduce gut epithelial production of IL-8 (102, 103).

Intestinal epithelial cytokine release prompted by viral infection can help clear infection or create pathology. Simian immunodeficiency virus infection of the intestinal epithelium of rhesus macaques induced IL-1β expression by Paneth cells before the induction of an antiviral IFN response. IL-1β expression was correlated with epithelial disruption characterized by the mislocalization and reduced expression of tight junction proteins, although these changes did not correspond to any aberrant responses to bacteria (104).

Multiple studies have documented the production of IFN-λ by virus-infected intestinal epithelial cells, although the ability of this cytokine to limit viral infection varied between studies (82, 84, 105). A possible explanation for these discrepancies may be found in the work of Hernández et al., which demonstrated that group 3 ILC-derived IL-22 amplified IFN-λ signaling in intestinal epithelial cells, and synergistic signaling by the two cytokines was necessary for a reduction in viral replication and optimal stimulation of IFN-induced gene expression (105).

### Dietary Modulation of Intestinal Epithelial Mediator Release

Diet has been implicated as a possible contributing factor to IBD; however, research has failed to identify the “ideal” anti-inflammatory diet for IBD patients (106). Nevertheless, recent studies have identified anti-inflammatory effects of specific dietary components on the intestinal epithelium. Pretreatment of Caco-2 cells with the plant-derived flavonoid cyanidin-3-glucoside (C3G) reduced TNF-α-induced gene expression of IL-8 and TNF-α. C3G also inhibited endothelial cell activation and subsequent leukocyte adhesion stimulated by coculture with TNF-α-stimulated Caco-2 cells (107). Similarly, treatment of Caco-2 cells with the dietary fiber guar gum increased expression of the suppressor of cytokine signaling-1 (SOCS-1) and reduced TNF-α-induced IL-8 expression. In addition, guar gum administration to mice with chemically induced enteritis reduced disease activity and pro-inflammatory cytokine expression in the small intestine concurrent with an increase in SOCS-1 protein (108).

## Concluding Remarks

Cytokines and chemokines are critical for intestinal epithelial homeostasis and responses to disease. The ability of cytokines to directly facilitate or restrict intestinal epithelial proliferation, apoptosis, and permeability makes them key players in the maintenance, or at times destruction, of the intestinal epithelial barrier. Furthermore, the release of cytokines and chemokines by the intestinal epithelium in response to pathogens, commensal organisms, interactions with other cell types, and dietary compounds allows these cells to have critical input into their microenvironment. Despite our frequent tendency to classify cytokines as either pro- or anti-inflammatory, we must realize that these labels fail to acknowledge the incredible diversity and situational basis of cytokine functions. While undoubtedly complex, the cytokine biology of intestinal mucosal immunology is a fascinating opportunity for investigations into both intestinal immunophysiology and potential translational approaches to modulate this physiology for much-needed novel therapies for intestinal disease.

## Author Contributions

CA, MM, and SD contributed to the development of the review topic. CA wrote the initial draft of the manuscript, and MM and SD critically reviewed and edited the manuscript for content.

## Conflict of Interest Statement

The authors declare that the research was conducted in the absence of any commercial or financial relationships that could be construed as a potential conflict of interest.
